# Phylogeny and phylodinamic of Hepatitis C in Italy

**DOI:** 10.1186/1471-2334-12-S2-S5

**Published:** 2012-11-12

**Authors:** Massimo Ciccozzi, Alessandra Lo Presti, Anna Rita Ciccaglione, Gianguglielmo Zehender, Marco Ciotti

**Affiliations:** 1 Department of Infectious, Parasitic and Immunomediated Diseases, National Institute of Health, Rome, Italy; 2 Department of Clinical Sciences Luigi Sacco, Section of Infectious Diseases, University of Milan, Italy; 3 Laboratory of Molecular Virology, Foundation Polyclinic Tor Vergata, Rome, Italy

## Abstract

Hepatitis C virus infection (HCV) is one of the most pressing health emergencies in the world with a global prevalence of about 170 million people chronically infected worldwide. In Europe, Italy has the highest HCV prevalence (3 - 4.4%) with peaks of 12.6 - 26% in Southern regions and the major islands. In Italy HCV genotype 1b prevails, and genotype 4 is mainly found in the south of the country where the prevalence is particularly high in regions such as Calabria.

Phylogenetics analysis is a molecular tool widely used to study rapidly-evolving RNA viruses that establish chronic infections such as HCV. Searching the scientific literature, it was found that thirty-nine genetic studies on HCV genotypes have been carried out in Italy between 1997 to 2012 years. However, phylogenetic analysis was performed only in fourteen out of thirty-nine HCV studies (36%) considered. Monitoring the genetic evolution of HCV is an essential step to control the local as well as global HCV epidemic and to develop efficient preventive and therapeutic strategies.

## Introduction

More than 170 million people are chronically infected with hepatitis C virus (HCV) worldwide. HCV is the leading cause of chronic liver disease, cirrhosis, and hepatocellular carcinoma in developed countries [1]. According to WHO estimates, at least 17 million people living in Eastern Mediterranean countries are HCV carriers emphasizing the importance of HCV infection in this part of the world [2].

Italy has the highest HCV prevalence (3 - 4.4%) in Europe. Higher prevalence rates (12.6 - 26%) have been reported in Southern regions and the major islands of Italy, which can be considered as hyper-endemic areas like other countries bordering the Mediterranean [3,4]. HCV has been divided into six major genotypes and a number of subtypes [5]. The distribution of HCV genotypes in Mediterranean countries has two main patterns. In Southern and Eastern Europe, the most common subtype is 1b, followed by genotypes 2 and 3.In North Africa, the most common subtype is 1b, whereas in non-Arab countries such as Turkey, and Israel, the most common subtype is 1b, followed by genotype 1a. In Iran subtype 1a is most common followed by genotypes 3a and 1b. In Arabic countries, such as Egypt, Lebanon, Syria, Saudi Arabia, and Kuwait, genotype 4 predominates [6].

In Italy, genotype 4 was found in the south of the country and the prevalence is particularly high in regions such as Calabria [3,7,8]. Retrospective information revealed that the majority of patients infected with genotype 4 live in small rural areas within the provinces of Reggio Calabria and Crotone where the prevalence, in patients from the Reggio Calabria Hospital, reached 8.4% [7]. Some observations suggest that genotype 4 might be endemic, particularly in such restricted areas of Calabria, as these areas are far away from the sea and important highways and likely remained isolated in the last two centuries. This review provides an overview of phylogenetic sequence studies performed on HCV viruses circulating in Italy.

## Phylogenetic methods

Phylogenetics is a branch of molecular biology that infers knowledge about taxonomy and evolution of species [9]. It is a powerful tool widely used in the study of rapidly-evolving RNA viruses that establish chronic infections such as HCV. In recent years a number of methods that infer phylogenetic trees have been introduced. These methods are based on genetic distances, evolutionary parsimony, Maximum-Likelihood and Bayesian theory [10-13]. Genetic distances and phylogenetic trees (coupled with a correct epidemiological design i.e., cross sectional studies), inferred via different sequence evolutionary models and model selection criteria, are normally used to assign the genotype [14]. In addition, phylogenetic and evolutionary methods have been widely used to define circulating recombinant forms (CRFs), discovering mosaics and complex form of the virus [15-20]. Coalescent theory and the molecular clock hypothesis are instead used to study the ancestral relationships of individuals sampled from a population (i.e., longitudinal studies) which can be inferred from a gene genealogy (phylogenetic tree) [15,17,20-23].

Phylodynamic inference has become a rapidly expanding field in the last years. Phylodynamic analysis employs coalescence theory which is usually used to investigate how the genealogy of a pathogen population, both at the intra- and inter- host level, is influenced by the interaction among pathogen’s demographic history, host immunological milieu and environmental/ecological factors [24,25].

**Figure 1:**
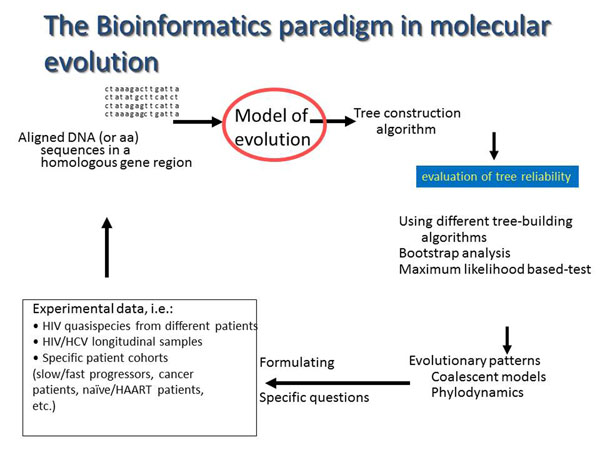
**Flow-chart representing the paradigm of phylogeny.** The major steps in phylogeny inference linking experimental design and data analysis are represented. This experimental design can be useful for HCV and /or other microorganism.

After the generation of the data set, the alignment with reference sequences, manual editing to delete “indels” (insertions/deletions), absence of contamination, and the determination of the phylogenetic signal is required. Phylogenetic and/or phylodinamic analyses represent the “core” of the data analysis and hypothesis testing. To test for the best substitution model, to infer phylogeny using different algorithms (e.g., genetic distance, Maximum-Likelihood, Bayesian methods), to test the trees reliability (e.g., by bootstrapping), are essential steps for evolutionary analyses [20,26].This hypothetical-deductive cycle is shown in Fig. 1.

## Italian studies

About thirty-nine genetic studies on HCV genotypes have been carried out in Italy from 1997 to 2012 years, with an interesting peak in 2003, Fig. 2.

**Figure 2:**
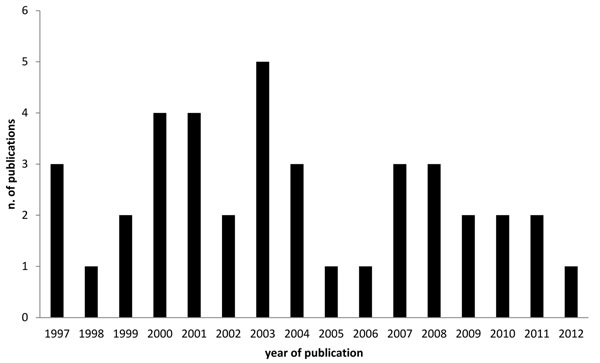
Number of publications on phylogeny of HCV by year of publication.

Maggi *et al.* in 1997 [27], reclassified serum samples from fifty Italian patients by a genetic analysis of NS5A gene region. The prevalent HCV genotype was subtype 2 c (90%). A high prevalence of HCV genotype 2c (22%) was detected in sera from 459 Italian patients by Biasin et al., [28]. The authors used a core-region amplification and hybridization with specific probes by DNA enzyme immunoassay.

Argentini et al*.*, in 2000 [29] studied the characteristics of genotype 4 subtype variability of HCV isolates circulating in Italy. The authors analyzed 736 HCV-RNA positive sera from 4 different regions of North, South Italy and Sardinia applying phylogenetic methods. Twenty-four out of 28 genotype 4 isolates (86%) were classified by phylogenetic analysis of E1 genome region as belonging to subtype 4d. An elevated level of viral heterogeneity was also observed among subtype 4 isolates identified in haemodialysis patients. This is probably related to a longer period of past endemicity of this genotype and to a high level of exposure to reinfections in particular categories of patients such as haemodialysis patients.

In 2000, Manzin et al., [30] studied the dynamics of the genetic diversification of HCV populations in perinatal infection. The authors showed that the variants detected in samples collected between birth and the third month of life were present in the HCV population of their mothers at delivery. They also observed that diversification of the intra host HCV population occurred from 6 to 13 months after birth. The authors suggested a dominant role of positive selection for amino acid changes in driving the pattern of genetic diversification of HCV populations, indicating that the intra host evolution of HCV populations is compatible with a Darwinian model system.

Gerotto et al., [31] used phylogeny to investigate the association between chronic HCV infection and development of mixed cryoglobulinemia type 2 (MC2), a lymphoproliferative disorder characterized by B cell monoclonal expansion and immunoglobulin M/k cryoprecipitable immunoglobulin production. A short sequence of the HCV E2 protein was investigated in 21 patients with HCV-related MC2 and in a control group of 20 HCV carriers without MC2. No mutations were observed in any of the sequences from control patients without MC2, and the difference between the 2 groups was statistically significant (P = 0.02). Analysis of 1345 HVR1 sequences retrieved from GenBank strongly supported the conclusion that the observed insertions and deletion represent a rare event in HCV-infected patients, suggesting that they are significantly associated with MC2.

In 2002 phylogenetic analysis was carried out by Larghi et al., [32] on HCV strains identified in 15 patients with acute hepatitis C (AHC) among 29 healthy volunteers participating in 2 consecutive pharmacokinetics studies. Analysis performed on the coding regions for the envelope glycoproteins E1 and E2 indicated a common source of infection.

Bagaglio et al. [33] correlated the degree of HCV variability with the response to anti-HCV treatment in HIV positive patients infected with HCV genotype 3a. In this study, 27 HIV positive and 5 HIV negative patients with HCV genotype 3a infection were treated with interferon-alpha-2b with or without ribavirin. Phylogenetic analysis of HCV paired serum samples at baseline and during treatment was used and revealed identical E2 sequence in 5/21 HIV positive NR patients, whereas 6 other sequences were strictly related to baseline E2 domain and the remaining 10 were divergent. The authors concluded that the genetic variability at baseline within the E2 region and NS5A protein of HCV 3a strain obtained from HIV positive and HIV negative patients was not associated with treatment response.

Silini et al., [34] investigated the sequence diversity of hypervariable region 1 of HCV in liver transplant recipients and correlated it with the recurrence of hepatitis. They considered twenty-six patients during a 2-year period; all had graft reinfection, and 14 patients developed hepatitis recurrence. Sequence variation was greater during the first 3 months post-OLT than during the remaining period. The authors observed that the genetic diversity within single samples was not related to hepatitis recurrence or other clinical features.

Abbate et al., in 2004 [35] investigated the HVR-1 quasispecies composition in patients chronically infected with HCV genotype 1b, treated with PEG-IFN-alpha-2b or STD-IFN-alpha-2b plus RBV. Evolution of viral quasispecies was analysed by constructing phylogenetic trees. The authors concluded that viral quasispecies surviving early therapeutic pressure are most likely able to give rise to either virus rebound or persistence at follow-up. In the same year Spada et al., [36] applied the minimum spanning tree (MST) model to identify the history of transmission of HCV infection in an outbreak involving five children attending a pediatric oncology-hematology outpatient ward from 1992 to 2000. They collected blood samples from all children, household contacts, and one health care worker positive for antibody to HCV. HCV-RNA detection was performed on these samples and smears of routinely collected bone marrow samples. Phylogenetic analysis of hypervariable region 1 of the E2 gene was performed for all isolates. The authors found that the MST model appears to be a useful tool in tracing the history of transmission in outbreaks of HCV infection.

In a paper by Minosse et al., [37] plasma and genital cytobrush samples from 85 HCV/HIV-co-infected women were analyzed by phylogenetic methods. The authors found that the composition of viral quasispecies was different in the 2 body compartments at both the nucleotide and amino acid level. HCV was detected in 27% of cytobrush samples. The data suggested that the genital and plasma quasispecies represent distinct subpopulations, which possibly reflects compartmentalized viral replication.

Ferraro et al., in 2008 [38] used the phylogenetic analysis to reconstruct the HCV genotype 1b dissemination in a small Sicilian town (Camporeale) with a 10.4% prevalence of HCV. In order to reconstruct the pattern of introduction and diffusion of HCV in this ecological niche, the NS5 genomic region of 72 HCV genotype 1 isolates (39 from Camporeale and 33 collected throughout Sicily) was sequenced. By applying Monte Carlo Markov simulation, the authors calculated that HCV was introduced between the end of the 1940s and the beginning of the 1950s. The phylogenetic analysis helped to conclude that in this small town few HCV native strains have been transmitted in a limited length of time, probably through iatrogenic routes, and then have not spread further.

In 2009 Sagnelli et al., [39] and co-authors described a phylogenetic analysis in three patients who, after symptomatic AHC, experienced subsequent episodes of HCV-related acute liver cell necrosis followed for three years. Genetic variations was investigated and the authors observed an association between the episodes of HCV-related acute liver cell necrosis after AHC and the different virological patterns, such as the establishment of a chronic HCV infection, a reactivation of an occult virus, or a reinfection by a different HCV genotype.

An interesting phylogenetic study was conducted by Bernini et al., in 2011 [40]. The authors investigated the within host dynamics of HCV quasispecies population in HIV-1 / HCV co-infected patients.

The intra-host evolution of the HCV heterogeneity in 8 co-infected subjects, selected from a cohort of 32 patients initiating HAART was also studied. The evolutionary rates were computed; moreover dated phylogenies and population dynamics were co-estimated by using a Bayesian Markov Chain Monte Carlo approach, and site specific selection pressures were estimated by Maximum Likelihood-based methods. A significant positive selection pressure was also detected in a half of the patients under HAART and in none of the group C controls. The authors described several sites under significant positive selection, located mainly in the HVR1 region. It was concluded that different forces, in addition to the selection pressure, drive an exceptionally fast evolution of HCV during HAART immune restoration.

Lastly, the recent study by Ciccozzi et al., [8] analyzed twenty-five HCV positive serum samples from Calabria classified as genotype 4. Phylogenetic analysis of the NS5B gene region reclassified the 25 samples as 19 (76%) genotype 4d, 2 (8%) genotype 4a, 1 (4%) genotype 1b, and 3 (12%) genotype 2a.

The Bayesian coalescent analysis was also applied and showed that the Italian 4d isolates collected in Calabria likely shared a common ancestor with other 4d isolates whose origin was traced back to 1943 (1913–1968). This finding suggests that spread of genotype 4d outside Africa started around this date, probably during the second World War. It was also observed, using a phylodinamic approach, the non gradual growth of the genotype 4d Italian epidemic which was maintained in a steady non-expanding phase until late 1970s, likely by sporadically acquired infections. After that, it grew exponentially between 1975 and 1990. The study shows that phylogenetic analysis can provide information about subtypes circulation and the use of a Bayesian coalescent method is necessary to date the origin and the dynamic of the epidemic.

## Conclusions

In Italy, HCV screening and surveillance is minimal at the national level. Most of the information is from local studies and it is difficult to extrapolate the data to the general population. It has been reported an age-related prevalence with most of the infections observed in the elderly, especially in central and south Italy, indicating that the risk of HCV infection has been greatest in the distant past (30-50 years ago) [41] due to unsafe medical practice such as injections with multi-use syringes. Currently the major risk factors are nosocomial and healthcare-associated transmission, while intravenous drug use is not a major problem in Italy. Genotype 1 is the most frequent (62%), followed by genotype 2 (27%), genotype 3 (7%) and genotype 4 (5%) [7].

HCV genotype determines the duration of antiviral therapy and predicts the response to treatment [42]. Therefore, sequence analysis plays a crucial role in the management of HCV infected patients. In addition sequencing is essential for monitoring epidemiological trends in various geographic areas and populations allowing the identification of HCV genotypes/subtypes circulating in the region of interest, and for the identification of resistance mutations in the genomic regions targeted by the antiviral therapy. Nevertheless, reviewing the Italian literature on this specific topic, phylogenetic analysis was performed only in fourteen out of the thirty-nine HCV genetic studies (36%) found using the key words ‘HCV phylogeny Italy”.

The analytical power of the phylogenetic, phylodinamic and Bayesian methods available today should prompt the researchers to use dataset as large as possible to monitor the epidemiological changes of HCV over time. Molecular characterization of several viral sub-genomic regions is advisable. Performing phylogenetic analyses only on the NS5A or B region may result in the lack of identification of novel subtypes or recombinants as shown by a recent study [43]. Therefore, whenever it is possible, combined sequencing and phylogenetic analysis should always be used in order to gain information about the starting of the epidemic, its spread and dynamics of viral strains.

Finally, phylogeographic methods can provide information about the spread of viral strains between different geographic regions [44].

Monitoring the genetic evolution of HCV using large dataset represents an essential strategy to control the local as well as global HCV epidemic and to develop efficient preventive and therapeutic strategies with a great impact in clinical practice.

## Competing interests

The authors declare that they have no competing interests.

## Declarations

Publication of this supplement was partly supported by an unrestricted grant provided by Roche. The articles were independently prepared by the authors with no input from Roche. Roche were not involved in selecting the articles for the supplement. The PEG-IFN-alpha-2b treatment mentioned in this article is produced by Roche.
